# Biochemical and genetic analysis of Ecm14, a conserved fungal pseudopeptidase

**DOI:** 10.1186/s12860-020-00330-w

**Published:** 2020-11-30

**Authors:** R. Christian McDonald, Matthew J. Schott, Temitope A. Idowu, Peter J. Lyons

**Affiliations:** Department of Biology, Andrews University, Berrien Springs, MI USA

**Keywords:** *Saccharomyces cerevisiae*, Fungi, Protease, Carboxypeptidase, Prodomain, Pseudoenzyme, Synthetic lethal assay, Cell wall, Extracellular matrix

## Abstract

**Background:**

Like most major enzyme families, the M14 family of metallocarboxypeptidases (MCPs) contains a number of pseudoenzymes predicted to lack enzyme activity and with poorly characterized molecular function. The genome of the yeast *Saccharomyces cerevisiae* encodes one member of the M14 MCP family, a pseudoenzyme named Ecm14 proposed to function in the extracellular matrix. In order to better understand the function of such pseudoenzymes, we studied the structure and function of Ecm14 in *S. cerevisiae*.

**Results:**

A phylogenetic analysis of Ecm14 in fungi found it to be conserved throughout the ascomycete phylum, with a group of related pseudoenzymes found in basidiomycetes. To investigate the structure and function of this conserved protein, His6-tagged Ecm14 was overexpressed in Sf9 cells and purified. The prodomain of Ecm14 was cleaved in vivo and in vitro by endopeptidases, suggesting an activation mechanism; however, no activity was detectable using standard carboxypeptidase substrates. In order to determine the function of Ecm14 using an unbiased screen, we undertook a synthetic lethal assay. Upon screening approximately 27,000 yeast colonies, twenty-two putative synthetic lethal clones were identified. Further analysis showed many to be synthetic lethal with auxotrophic marker genes and requiring multiple mutations, suggesting that there are few, if any, single *S. cerevisiae* genes that present synthetic lethal interactions with *ecm14*Δ.

**Conclusions:**

We show in this study that Ecm14, although lacking detectable enzyme activity, is a conserved carboxypeptidase-like protein that is secreted from cells and is processed to a mature form by the action of an endopeptidase. Our study and datasets from other recent large-scale screens suggest a role for Ecm14 in processes such as vesicle-mediated transport and aggregate invasion, a fungal process that has been selected against in modern laboratory strains of *S. cerevisiae*.

**Supplementary Information:**

The online version contains supplementary material available at 10.1186/s12860-020-00330-w.

## Background

Most major enzyme families contain inactive enzyme homologs, or pseudoenzymes. Pseudoenzymes are characterized by highly conserved domains that are structurally similar to their catalytically active homologs, but with substitutions within key motifs suggesting a loss of catalytic function [1, 2]. Research is just now beginning to show the large part that these proteins play in biology; they are much more than just innocent bystanders [3]. Pseudoenzymes have been shown to play key roles as allosteric modulators and non-catalytic competitors in a variety of signal transduction pathways [2].

Enzyme families in which pseudoenzymes have been particularly well-characterized include kinase and protease families [4–6]. For example, within kinase families, the pseudokinase FAM20A has been shown to allosterically activate its homolog, FAM20C, to enable increased phosphorylation of secreted enamel proteins [7]. Another pseudokinase, vaccinia-related kinase 3 (VRK3), also works through allosteric activation, but the activation of a phosphatase, resulting in inhibition of extracellular-regulated kinase (ERK) activity and its proliferative effects [8]. Within protease families, a number have also been shown to be pseudoenzymes. For example, the pseudoenzyme iRhoms regulate proteasomal degradation and are necessary for the translocation of the ADAM17 metalloprotease so that it can cleave the TNFalpha precursor [6, 9]. Inactive ADAMs 11, 22, and 23 serve as receptors for the secreted neuronal protein, LGI1; when ADAM22 is mutated it leads to a form of epilepsy [10, 11].

The M14 family of metallocarboxypeptidases (MCPs) is involved in trimming C-terminal amino acids from substrate proteins and peptides, including neuropeptides, clotting factors, and dietary peptides [12, 13]. This family of proteases has been studied for nearly 100 years due to the abundance of some members within the pancreas; as such, we know a great deal about the catalytic and regulatory mechanisms of these enzymes [14, 15]. The M14 MCP family is typically divided into three subfamilies, based on structural similarities. The pancreatic enzymes are members of the A/B subfamily, most of which are secreted and activated upon removal of a prodomain [16–18]. A number of MCPs, of the N/E subfamily, are either secreted or function within the secretory pathway, where they function in the maturation of a variety of peptide hormones and neuropeptides [19–21]. The remaining members of this family are the cytosolic carboxypeptidases, which are important in regulating the activity of microtubules within the cytoplasm [22–24].

**Fig. 1:**
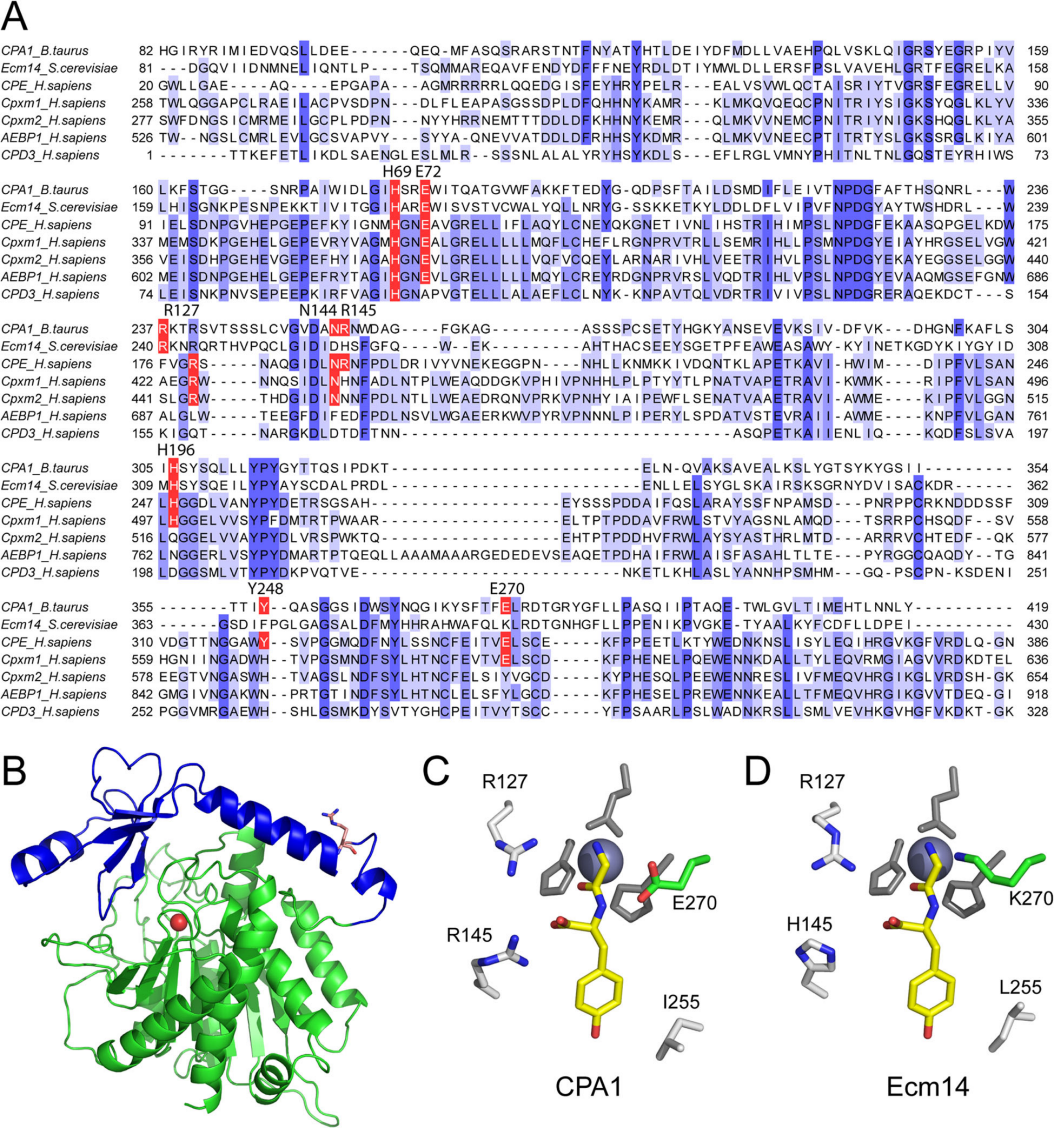
The M14 metallocarboxypeptidase family includes several predicted pseudopeptidases. **a** The amino acid sequences of two active M14 metallocarboxypeptidase enzymes, CPA1 and CPE, were aligned with the sequences of several predicted pseudoenzymes of the M14 family using Clustal Omega. The sequences of the carboxypeptidase domains only are shown. Amino acids considered critical active site residues within M14 carboxypeptidases are shown in red, with the corresponding numbering from mature bovine CPA1 shown above. Blue background shading indicates increasing levels of sequence conservation from light to dark. Numbers on left and right indicate amino acid number within the complete protein. **b** A three-dimensional model for *S. cerevisiae* Ecm14, with the prodomain shown in blue and the carboxypeptidase-like domain shown in green. A coordinated zinc is modeled as a red sphere, and the arginine at which a tryptic-like enzyme may cleave is shown in stick representation. **c** The active site of bovine CPA1 is compared with the corresponding amino acids within *S. cerevisiae* Ecm14 (**d**)

Included within the approximately 23 members of this enzyme family in mammals are several predicted pseudoenzymes, carboxypeptidase X1 (Cpxm1) [25], carboxypeptidase X2 (Cpxm2) [26], AE-binding protein 1 (Aebp1) [27, 28], and the third domain of carboxypeptidase D (CPD) [29] (Fig. 1a). While Cpxm1, Cpxm2, and Aebp1 are all closely related and have been found in recent years to be involved in connective tissue biology [30, 31], the function of the third domain of CPD is unclear at this time. It is not unimportant, however, as it has been shown that this third domain of CPD is necessary for full rescue of an inviable Drosophila CPD deletion [32].

The yeast *Saccharomyces cerevisiae,* a common model for eukaryotic cell biology, encodes only one member of the M14 MCP family within its genome (Fig. 1b). This gene is named extracellular mutant 14 (*ECM14*; *YHR132c* in the systematic nomenclature) due to its identification in a screen for genes involved in cell wall integrity, specifically a sensitivity to calcofluor white [33]. The Ecm14 protein is structurally similar to the A/B subfamily of MCPs, and is predicted to be a pseudoenzyme, as a number of active site residues critical for function of MCPs are substituted; residues involved in the coordination of the C-terminus of a substrate protein or peptide are substituted (N144D and R145H, using bovine CPA1 numbering that is typically used in the field), and, most notably, the glutamic acid that serves in acid-base catalysis, at position 270 in bovine CPA1, is replaced with a lysine (Fig. 1a, d).

The specific function of Ecm14 is unknown, as is its conservation throughout the fungal kingdom. In the current study, we set out to characterize the function of Ecm14, to shed further light on the function of this protein and the function of other pseudoenzymes within this MCP class of enzymes. We show that Ecm14 is conserved across a wide range of ascomycete fungi and can be processed by trypsin-like enzymes to remove the prodomain. Using yeast genetics we show that few, if any, single yeast genes in *S. cerevisiae* share synthetic lethal interactions with the gene deletion, *ecm14*Δ, suggesting either the presence of many redundant pathways or a function unnecessary within the context of typical laboratory strains of *S. cerevisiae*.

## Results

Ecm14 is the only member of the M14 metallocarboxypeptidase family found in the *S. cerevisiae* yeast. Ecm14 appears to be a pseudoenzyme, exhibiting substitutions at several key catalytic residues (see Fig. 1a,d). We set out to determine the function of Ecm14 through the use of biochemical and genetic approaches.

### Phylogenetic analysis of fungal Ecm14

**Fig. 2:**
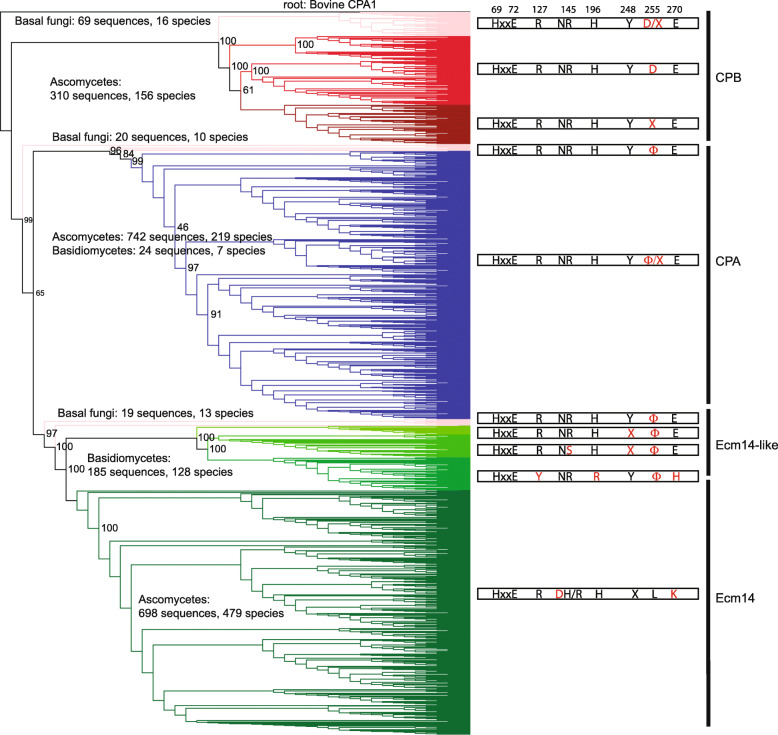
Ecm14 is conserved in Ascomycota, but not in other fungal phyla. BLASTp was performed with bovine CPA1 as the query sequence and all fungal sequences as the search set. Two thousand sixty-six sequences of 150 amino acids or longer that contained the majority of the carboxypeptidase domain were identified using Clustal Omega and the best maximum likelihood phylogenetic tree inferred using IQ-TREE. Branch support was determined by 1000 ultrafast bootstrap replicates, and bootstrap support values are shown at key nodes. The resulting tree contains three predominant groups consistent with CPB-like, CPA-like, and Ecm14-like properties. The identities of these groups are demonstrated by the active site residues found in all metallocarboxypeptidases (shown in black on the right) and unique variants (red; particularly at position 255) that often define the subclass. On the phylogenetic tree, sequences from basal (monokaryotic) fungi are shown in pink. CPB sequences from ascomycetes are shown in red, with co-clustering sequences lacking the required Asp at position 255 shown in a darker red. CPA-like sequences, mostly from ascomycetes, are shown in blue. Ecm14 sequences, all from ascomycetes, are shown in dark green. A sister clade of Ecm14-like proteins from basidiomycetes, and with unique and conserved ‘active’ site residues, is shown in lighter shades of green. Ecm14 proteins are defined by the presence of N144D and E270K substitutions

We first took a bioinformatics approach to understand the role of Ecm14. All fungal proteins within the NCBI database were searched (June 2019) using BLASTp for homology to bovine CPA1. This resulted in a dataset of 2066 sequences, after the deletion of sequences shorter than 150 amino acids or those lacking the majority of the carboxypeptidase domain. A phylogenetic tree was inferred by maximum likelihood using IQ-TREE with 1000 bootstrap replicates, and the consensus tree presented with bovine CPA1 as the root of the tree (Fig. 2; see also Additional files 1 and 2 for .nexml phylogenetic tree data files for both the consensus and maximum likelihood trees).

Protein sequences generally fell into three large groups, CPA-like, CPB-like, and Ecm14-like proteins (Fig. 2). Strong bootstrap support was obtained for most major branchpoints of this tree, except for the node between the major CPA and Ecm14 groups. This likely reflects weak support for the position of the CPA and Ecm14 sequences from the basal fungi. This ambiguity is further supported by the fact that the active site residues from these basal fungi enzymes are not at all different, all exhibiting CPA-like character. CPA-like enzymes are predicted to cleave aliphatic or aromatic C-terminal amino acids due to a hydrophobic amino acid at the bottom of their specificity pocket (residue 255 in bovine CPA1), while CPB-like enzymes are predicted to cleave basic C-terminal amino acids with an acidic amino acid, often aspartic acid, at their residue 255 equivalent [18].

Fungal proteins identified within the CPB clade often had an Asp at position 255; however, a lot of variation was seen at this position, as nearly every amino acid was represented. Similar results were observed in the CPA clade; while most proteins had a hydrophobic amino acid at position 255, many other amino acids were found to occupy this position also. Notably, no CPB sequences were found in Basidiomycota, and only 24 of 766 sequences in the CPA group were from Basidiomycota. Because a number of sequences from each of these three major groups (CPA-like, CPB-like, and Ecm14-like) were identified from organisms traditionally called the basal fungi or the monokaryotic fungi (eg. Chytridiomycota and Zygomycota) [34], it seems most likely that the CPA and CPB genes have been lost in most members of the basidiomycete clade.

The Ecm14 clade was the largest of the three, similar to the CPA clade in number of sequences but far exceeding it in terms of species representation (479 species for Ecm14 versus 219 species for CPA; Fig. 2). All Ecm14 sequences were derived from ascomycete fungi and were strictly defined by the presence of Asp at position 144 and Lys at position 270. Active metallocarboxypeptidases have a conserved Asn at position 144, which is involved in substrate binding, and a critical Glu at position 270 which serves as a proton donor and acceptor in general acid-base catalysis [14].

A large group of CP-like proteins from Basidiomycota were identified as a sister clade to Ecm14, in which the active MCP Asn144 was conserved. However, in about half of these sequences, Arg145 was replaced with a Ser, likely resulting in the inability to bind the carboxyl group of a substrate. In the other half of these sequences, three active site substitutions were found: Arg127Tyr, His196Arg, and Glu270His (Fig. 2). Arg127 typically functions in binding of the scissile carboxyl group during cleavage, while His196 coordinates the zinc ion cofactor of active enzymes and, of course, Glu270 is necessary for acid-base catalysis. The presence of substitutions at these important residues suggests that these proteins are also inactive enzymes.

Altogether, these results suggest that Ecm14 is a widely-distributed protein within the ascomycete branch of the fungal kingdom with unique active site residues that are conserved and thus likely to be functional.

### Ecm14 purification and biochemical analysis

**Fig. 3:**
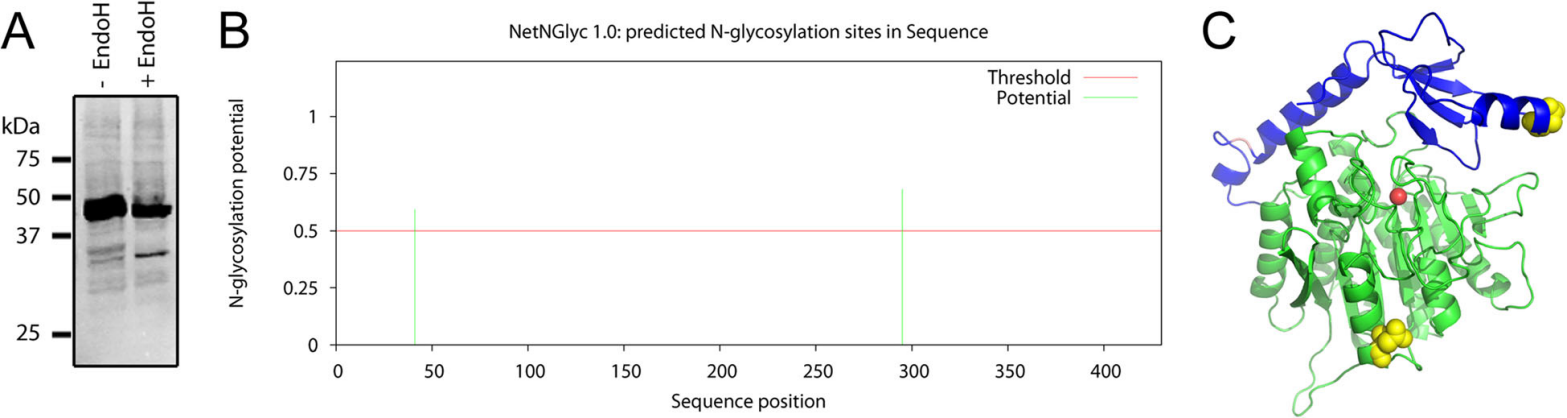
Ecm14 is N-glycosylated and processed by an endopeptidase in *S. cerevisiae*. **a** Yeast extracts that overexpressed Ecm14-His6 were incubated with endoglycosidase H (EndoH) and analyzed by western blotting with an anti-His6 antibody. ProEcm14 observed as a broad band at 45 kDa (likely two bands) collapses to one thin band with EndoH, and mature Ecm14 observed as two bands at 35 kDa collapses to one. **b** NetNGlyc 1.0 predicts there to be two N-glycosylation sites within Ecm14, on asparagines at positions 41 within the prodomain and 295 within the carboxypeptidase domain. **c** These asparagines, shown as yellow spheres, are both located on the surface of the Ecm14 homology model. The prodomain of Ecm14 is shown in blue, the carboxypeptidase-like domain in green, and a coordinated zinc is modeled as a red sphere

In order to determine whether Ecm14 was functional as a carboxypeptidase enzyme or as a pseudoenzyme, we attempted purification by metal-affinity chromatography. Histidine-tagged Ecm14 was expressed in its native environment, the yeast *S. cerevisiae,* under the control of a galactose-inducible promoter from the plasmid pEMBLyEx4. This resulted in abundant Ecm14-His6 expression, typically observed as a doublet approximately 45 kDa in size (pro-Ecm14) and another doublet at 35 kDa (mature Ecm14), as detected by anti-His6 western blotting (Fig. 3a). The two doublets were due to N-glycosylation of Ecm14, confirmed by a reduction of doublets to singlets upon incubation with EndoH (Fig. 3a). N-glycosylation was predicted at two sites by NetNGlyc, both sites found on the surface of Ecm14 (Fig. 3b,c).

Ecm14-His6 protein overexpressed in yeast was mostly insoluble and was not able to be purified even following denaturation in urea or guanidine. In contrast, when Ecm14-His6 was expressed in the Sf9 insect cell system using a baculovirus expression vector, about 50% of Ecm14-His6 protein was secreted into the media in soluble form by 3 days post-infection, typically as a doublet at 45 kDa (see Additional file 1: Figure S1A). A small amount of this 45 kDa protein was processed to produce a 35 kDa protein in Sf9 cells (see Additional file 3: Figure S1A), suggesting the removal of the prodomain. Addition of trypsin or chymotrypsin to conditioned media converted the large majority of 45 kDa protein to the 35 kDa form, and also greatly enhanced the purification of Ecm14-His6 by metal-affinity chromatography (see Additional file 3: Figure S1B-D).

**Fig. 4:**
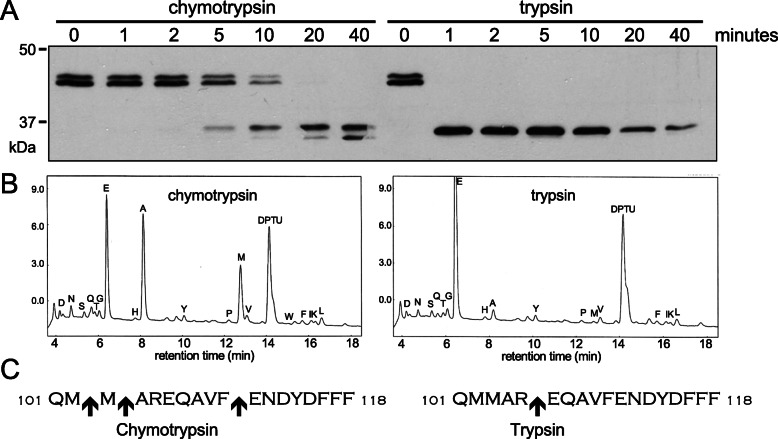
The prodomain of Ecm14 can be cleaved by chymotrypsin and trypsin enzymes. **a** Media containing proEcm14-His6 secreted from baculovirus infected Sf9 cells was digested with chymotrypsin or trypsin for the indicated times and analyzed by Western blotting with an anti-His6 antibody. **b** Following this digest, the resulting mature protein was purified by metal-affinity chromatography and N-terminally sequenced by Edman degradation. Results of the first round of sequencing are shown. **c** Sites of cleavage, as shown by Edman degradation, within the region of Ecm14 linking the prodomain and the carboxypeptidase-like domain, are indicated by arrows

The observation that Ecm14 could be processed into a smaller form by endopeptidase digestion both in vivo and in vitro suggested possible prodomain removal, and that this cleavage may be necessary for full activity of Ecm14. To investigate this maturation process further, Ecm14-His6 within conditioned Sf9 media was digested with trypsin and chymotrypsin. Digestion with 5 μg/ml chymotrypsin showed the reaction to be complete after 40 min at room temperature, with at least two products observed at about 35 kDa. Optimal cleavage by 5 μg/ml trypsin was observed after only 1–5 min, with one distinct band observed at 35 kDa (Fig. 4a). To identify the sites of cleavage, Edman degradation was performed on purified Ecm14-His6 following cleavage by either chymotrypsin or trypsin. It was found that chymotrypsin cleaved at 3 locations within the linker region between the prodomain and the carboxypeptidase domain, while trypsin cleaved only once, C-terminal to an arginine in this same region (Fig. 4b, c, see Additional file 3: Figure S2). The much cleaner and more rapid digestion by trypsin suggested that a trypsin-like enzyme might be important for full Ecm14 activity in vivo.

Purified Ecm14-His6 was incubated with standard carboxypeptidase substrates (3-(2-Furyl)acryloyl-Phe-Phe, -Phe-Ala or -Glu-Glu) at 0.5 mM and at a range of pH values from 5.5 to 7.5. No activity was detected under any of these conditions, even though the prodomain was no longer present.

### Biological function of Ecm14 through targeted genetic analyses

A number of large-scale screens in yeast have suggested possible functions for Ecm14. Ecm14 was first identified in a large screen searching for mutations leading to sensitivity to calcofluor white, suggesting a cell wall defect [33]. We have not been able to clearly replicate this phenotype. While a slight sensitivity to calcofluor white was observed upon deletion of the *ECM14* gene (*ecm14*Δ) in the BY4741 *MAT*a strain (see Additional file 3: Figure S3A), an *ecm14*Δ deletion in the same strain stored independently in another lab did not result in the same sensitivity (see Additional file 3: Figure S3B).

Hillenmeyer et al. attempted to tease out the functions of nonessential yeast genes through a large-scale screen in which they measured the fitness of yeast mutants upon incubation with a variety of chemicals or in various environmental conditions [35]. Decreased fitness was observed for *ecm14*Δ cells under a number of conditions. We incubated wild-type and *ecm14*Δ yeast in YPD media supplemented with many chemicals that were shown by Hillenmeyer et al. to effect fitness, including paraquat (5 mM), miconazole nitrate (25, 50, and 100 µM), alverine citrate (500 µM), hydrogen peroxide (2 and 5 mM), D-sorbitol (0.75 and 1.5 M), mercury (II) chloride (15.6 and 62.5 µM), and lithium chloride (150 and 300 mM). Of these, a small and reproducible effect was observed for lithium chloride only, that being a small increase in *ecm14*Δ cell growth rate compared to wild-type cells (see Additional file 3: Figure S3C).

Other reports have described a number of genetic synthetic lethal phenotypes. For example, yeast with *arp1*Δ deletions were shown to be inviable together with an *ecm14*Δ mutation [36]. Our attempts to replicate this result also came up empty-handed. Our BY4741a yeast with both *ecm14*Δ and *arp1*Δ deletions remained viable.

### Biological function of Ecm14 through synthetic lethal genetic analysis

**Fig. 5:**
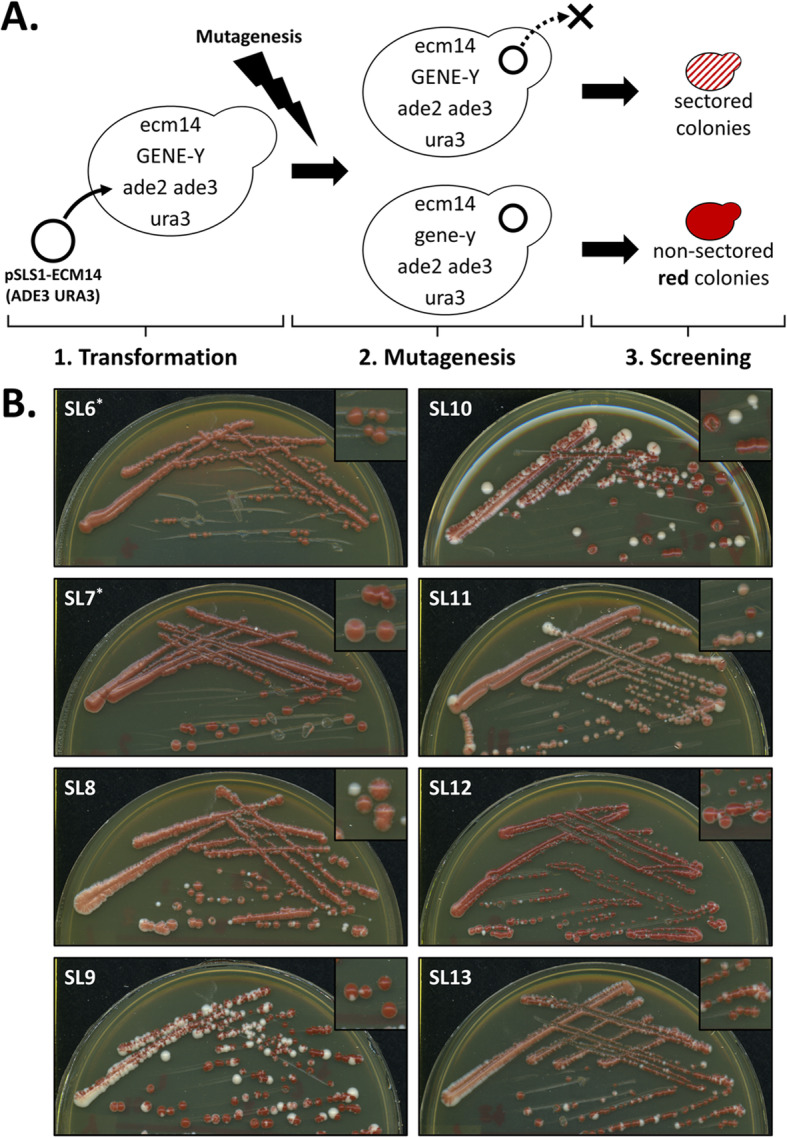
The synthetic lethal assay used to identify novel mutant yeast whose survival was dependent on the *ECM14* gene. **a** (1) Wild-type *ECM14* was first moved from the yeast genome into the pSLS1 vector (pSLS1-ECM14). (2) Random genomic point mutations were introduced throughout the yeast genome using ethyl methansulfonate (EMS). (3) Colonies dependent on *ECM14* were identified as non-sectored red colonies. **b** Putative synthetic lethal mutants that initially produced the desired non-sectored red phenotype were re-streaked for single colonies on galactose media to evaluate the development and maintenance of the red phenotype. Asterisks indicate colonies that were screened on media containing Ura + CFW

In lieu of any clearly identifiable phenotype for *ecm14*Δ cells, we sought to identify the function of *ECM14* through a synthetic lethal approach, in which the lethality of two gene mutations is rescued by expression of one wild-type gene from a plasmid. While many genes putatively synthetic lethal with the *ecm14Δ* gene have been listed in the yeast database (yeastgenome.org) from large genome-wide screens in recent years, many of these putative genetic interactions do not exceed default Pearson correlation coefficient thresholds (see thecellmap.org), and so we reasoned that a synthetic lethal experiment targeted specifically at *ecm14*Δ would provide better-validated data. Therefore, *ecm14*Δ cell suspensions, transformed with pSLS1-ECM14, were treated with ethyl methanesulfonate (EMS) and plated on rich media containing galactose to destabilize the pSLS1-ECM14 plasmid (see Additional file 3: Figure S4 for a map of this plasmid). Those yeast acquiring secondary mutations requiring the *ECM14* gene for viability would maintain the plasmid and would be identified due to red pigmentation produced by the *ADE3* gene on the plasmid (Fig. 5a). Yeast not requiring the plasmid for viability would rapidly lose it, resulting in white or sectored colonies. YPD plates were used as controls to determine the effectiveness of EMS mutagenesis and to tune EMS exposure time. An EMS exposure time of 45 min was determined to be effective at generating the ideal cell death (~ 40%; (Winston, 2008)) in the yeast strains used in this study (see Additional file 3: Figure S5).

Because the pSLS1-ECM14 plasmid contains two additional yeast genes (*ADE3* and *URA3*), this approach could also identity false-positive synthetic lethals caused by a dependency on either the plasmid-borne *ADE3* or *URA3* genes. Effectively silencing either the *ADE3* or *URA3* gene could potentially improve the sensitivity of the synthetic lethal screen and decrease the number of false-positives to be subsequently evaluated. Because the *URA3* gene was not involved in the synthetic lethal screen, a modification was made in later experiments in which YPGal media containing extra uracil (0.001%) was used to decrease undesirable *URA3* false-positives. In addition, a modified YPGal medium containing 20 μg/ml calcofluor white was also used for screening EMS-treated cells. Exposure to calcofluor white (CFW) amplifies the effect of mutations in genes involved in cell wall architecture and remodeling [33], and therefore it was thought that CFW might increase the sensitivity of the synthetic lethal screen to novel mutations in genes functionally related to *ECM14*. An active, plasmid-borne *ADE3* gene was required for the *ADE3*/*ADE2* color assay essential to the synthetic lethal screen. Therefore, modifications to decrease *ADE3* false-positives were not made.

Approximately 27,000 EMS-treated *ecm14*Δ [pSLS1-ECM14] yeast colonies were screened for novel plasmid dependencies on galactose media (either YPGal, YPGal+CFW, or YPGal+Ura + CFW). Three rounds of EMS mutagenesis were performed, with each yielding approximately 9000 screenable EMS-treated yeast colonies. Colonies of interest were those that produced the desired non-sectored red phenotype on these media. These non-sectored red colonies of interest were re-streaked for single colonies on fresh media to further evaluate the development and the maintenance of the desired non-sectored red phenotype (Fig. 5b). The first round of EMS mutagenesis yielded five putative synthetic lethal mutants (SL1–5) derived from YPGal. The second round yielded two putative synthetic lethal mutants derived from YPGal+Ura + CFW (SL6–7) and seven from YPGal (SL8–14). The final round yielded eight putative synthetic lethal mutants derived from YPGal+Ura and YPGal+Ura + CFW (SL15–22). SL10, 11, and 13 were unique in that they did not retain their red color when plated onto YPGal containing calcofluor white. Some colonies of interest, particularly SL 9, 10, and 13, that initially produced the desired non-sectored red phenotype on these media following EMS treatment, produced a mosaic phenotype when re-streaked on galactose media, suggesting that they may not be true synthetic lethals.

Additional rounds of growth on both SC-Ura and SC + 5FOA were attempted to test for true synthetic lethal mutants caused by novel secondary mutations or for potential false-positives caused by reversions of marker genes. Counterselection against the plasmid-borne *URA3* gene using 5FOA should result in plasmid loss and, thus, cell death for true synthetic lethal mutants. This test was performed on the first five putative synthetic lethal mutants (SL1–5; see Additional file 3: Figure S6). Although these five putative synthetic lethal mutants seemed to retain the desired phenotype when subsequently re-plated/spotted on YPGal, all five were unable to grow appreciably on any SC-based media. However, because the EMS-treated white non-synthetic lethal colony (*ecm14*Δ) and untreated control strain (*ecm14*Δ [pSLS1-ECM14]) were able to grow on SC-based media, the apparent lack of growth of these synthetic lethal mutants seemed to be the result of secondary mutations conferring significant fitness deficiencies on SC-based media.

**Fig. 6:**
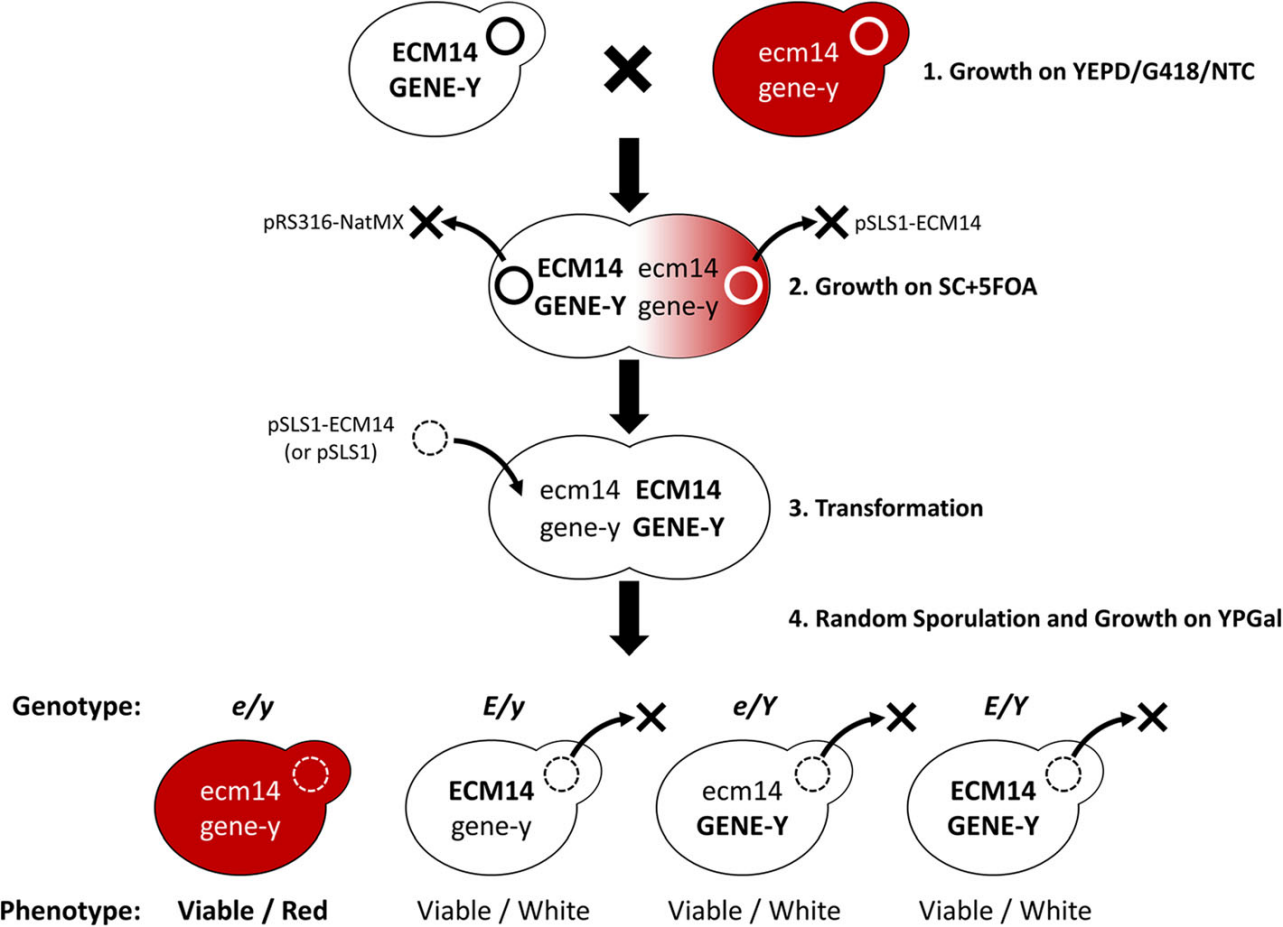
The functional rescue assay workflow. (1) A diploid yeast strain is produced by crossing a wild-type haploid [pRS316-NatMX] strain with a haploid synthetically lethal strain (gene-y, *ecm14::KanMX*). Diploids are selected for by growth on YPD/G418/NTC. (2) Growth on SC + 5FOA allows for selection against the now unnecessary pRS316-NatMX and pSLS1-ECM14 plasmids. (3) Cells are transformed with the pSLS1 or pSLS1-ECM14 plasmids followed by (4) random sporulation and germination of these haploid spores on YPGal

Synthetic lethal mutants were further analyzed to confirm synthetic lethality with *ecm14*Δ (Fig. 6). Mutants were crossed with wild-type yeast of an isogenic background, containing a plasmid (pRS316-NatMX) allowing growth on nourseothricin, and heterozygous diploids were selected by growth on YPD + G418 + nourseothricin. Diploid colonies were grown on SC + 5FOA to eliminate plasmids, and then transformed with pSLS1 or pSLS1-ECM14. Sporulation of diploids was performed and random spores germinated on YPGal plates. The percentage of unsectored red colonies was determined, and compared to expected segregation of the phenotype. If only two mutations were required for the red synthetic lethal phenotype (ie. the *ecm14*Δ mutation and mutation of one additional gene by EMS), then we would expect approximately 1/4 of spores derived from a heterozygous diploid to be red. If two EMS mutations were required for synthetic lethality, then approximately 1/8 of spores would be red, and so on.

An average of 204 germinated spores were analyzed per synthetic lethal clone. Nine of 16 synthetic lethal clones were rescued by the pSLS(−) plasmid itself, indicating synthetic lethality with another element on the plasmid (Table 1). In these cases, the fraction of total colonies being red suggested that 1–3 EMS mutations were required for the synthetic lethality. Very few, if any, red colonies were observed for the remaining seven clones, suggesting that these were either not true synthetic lethals, or that four or more EMS mutations were required to generate the synthetic lethality, and, therefore, that useful genetic information would be difficult to identify (Table 1).

**Table 1:** Analysis of isolated synthetic lethals

Name	pSLS1(−) rescue (% red colonies)	pSLS-ECM14 rescue (% red colonies)	# of causative mutations	Possible causative mutation^a^
WT	0	0	–	–
SL1	3.9	2.7	3	ND
SL2	0	0	≥4	ND
SL4	0	0	≥4	ND
SL6	16.8 (1/6)	20.6 (1/5)	1	SHM2 D219N (*ade3* SL)
SL7	21.0 (1/5)	18.5 (1/5)	1	SHM2 D219N (*ade3* SL)
SL8	7.7	0	3	
SL9	0	1.5	≥4	ND
SL10	0	0	≥4	ND
SL11	10.5 (1/10)	10.6 (1/10)	2	SHM2 G403R (*ade3* SL)
SL12	0	0	≥4	
SL13	0	0.8	≥4	SHM1 P252S (*ade3* SL)
SL14	6.9	8.9	2	SHM2 G252D (*ade3* SL)
SL15	15.0	9.1	2	ND
SL18	0	0	≥4	ND
SL20	5.0	0	3	ND
SL21	15.1	7.6	2	ND

^a^ *ND* not determined

### Identification and analysis of ECM14 interacting genes

**Fig. 7:**
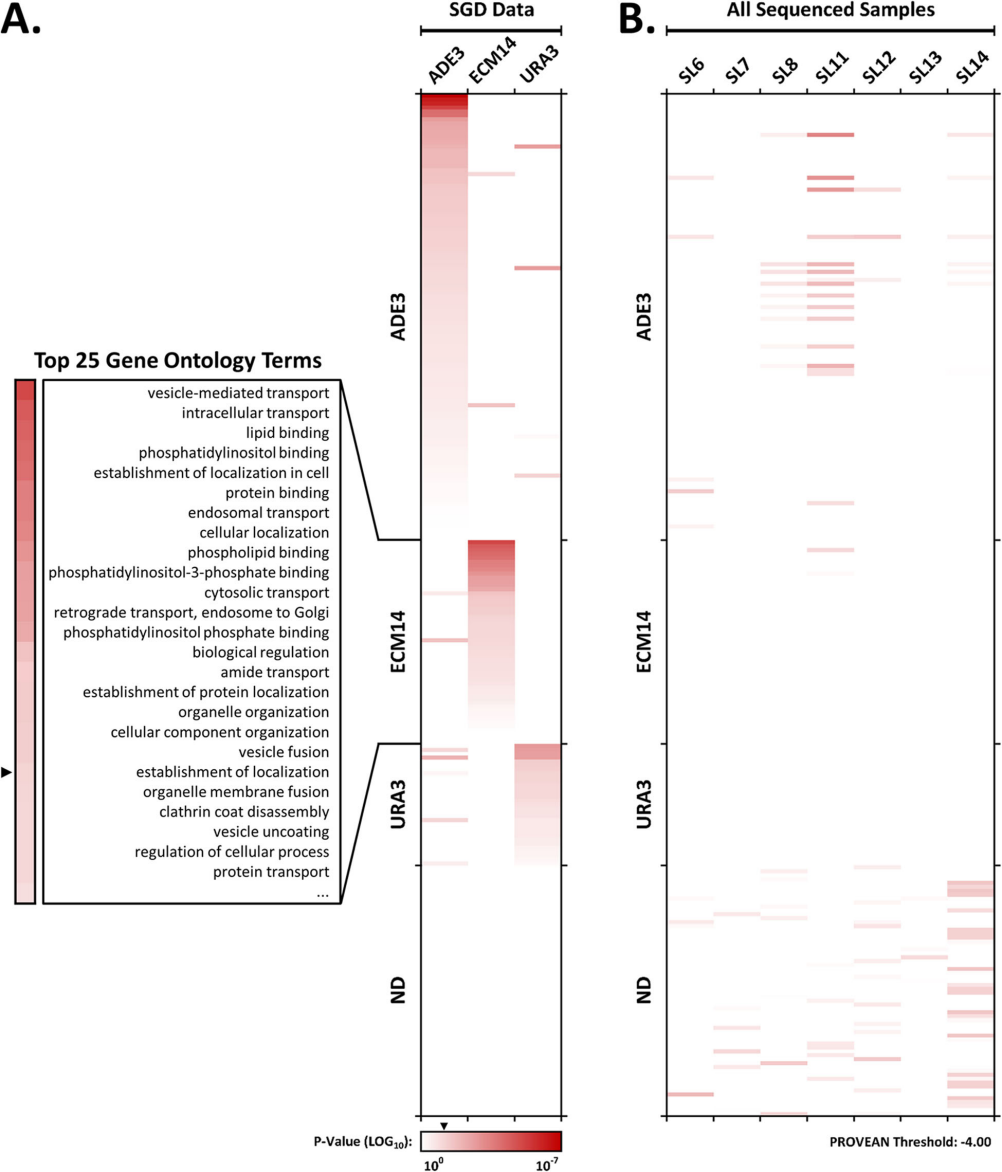
Gene Ontology (GO) analysis of genetic interactors. **a** Genes annotated by the *Saccharomyces* genome database (SGD) as interacting either physically or genetically with *ADE3*, *ECM14*, or *URA3* were analyzed by Gene Ontology (GO). Little overlap is observed between GO terms for these three genes. The heatmap represents *P*-values for enrichment of genes within particular GO classifications. Arrowhead next to heatmap scale bar indicates 0.05. Top 25 GO terms are shown for *ECM14* only. **b** GO analysis for genes identified by whole-genome sequencing as being mutated in the indicated synthetic lethals, and for which PROVEAN analysis surpassed a threshold of − 4.0. GO terms are mapped to those identified in the SGD for *ADE3*, *ECM14*, and *URA3* interactors. All others are listed at ND, not determined

*ECM14* interacting genes were analyzed in two ways. First, all genes reported within the *Saccharomyces* genome database (SGD) to interact either physically or genetically with *ECM14* were submitted for Gene Ontology (GO) analysis. The top reported GO function and process terms, found to be enriched in this gene list compared with all yeast genes (*p* < 0.05) included terms such as *vesicle-mediated transport, lipid binding, and organelle organization* (Fig. 7). About 40% of interacting genes shared the term *endomembrane system*. These terms are consistent with a secreted or secretory role for *ECM14* in cell wall maintenance. Genes reported in the SGD to physically or genetically interact with *ADE3* or *URA3* were also assessed by GO analysis. This GO analysis was performed in order to compare *ADE3* and *URA3* interacting genes with those mutated in our synthetic lethal assay, to further determine likely relevance.

Seven putative synthetic lethal mutants (SL6, SL7, SL8, SL11, SL12, SL13, and SL14) were selected for whole-genome sequencing using the Illumina MiniSeq platform. Clones were chosen for a diversity of red color phenotypes prior to the above complementation analysis. Sequencing resulted in 30-50x coverage (Table S1) and, after elimination of noncoding and silent variants when compared to the WT sequence, 46–123 variants were identified for each synthetic lethal clone. Because this was too many to be useful, further analysis was performed in PROVEAN to identify those variants likely to be deleterious, using a stringent cutoff of − 4.0. GO analysis was performed on these gene lists. For all clones except SL7 and SL13, some enrichment (although not always reaching significance, *p* < 0.05) was found for GO terms associated with *ADE3* interacting genes. Very few of the GO terms identified for remaining synthetic lethal gene mutations reached a *p* < 0.05 level of significance. In some cases, missense mutations were identified in genes known to be synthetic lethal with *ade3*. For example, both SL6 and SL7 presented very consistently red synthetic lethal colonies; a mutation in the SHM2 gene resulting in a D219N amino acid change was identified in both (Table 1). Likewise, SL11 and SL14 contained mutations in the SHM2 gene, while SL13 contained a mutation in the SHM1 gene. Both the SHM1 and SHM2 genes are reported in the SGD to be synthetic lethals of the *ade3* gene. These results are consistent with the rescue of SL6, SL7, SL11, and SL14 by the pSLS(−) plasmid, suggesting that the mutations in these clones leading to synthetic lethality are mutations within the *ADE3* or *URA3* pathways rather than within *ECM14* interacting genes.

Two of the synthetic lethal clones that were sequenced, SL12 and SL13, were not rescued by the pSLS(−) plasmid (see Table 1), and the results of this rescue experiment suggested that 4 or more mutations were involved in this phenotype. A careful GO analysis of the 36 genes mutated in SL12 and SL13 having PROVEAN scores of less than − 4.0 resulted in the gene ontology terms (*p* < 0.05) *lyase activity* and *adenylate cyclase activity*, in addition to the more general terms *small molecule binding, cofactor binding*, and *catalytic activity*. The five genes fitting the categories of lyase and adenylate cyclase activities were *PXP1*, encoding a peroxisomal protein, *SOG2*, encoding a signaling protein involved in cell morphogenesis and cytokinesis, *ILV3*, encoding an enzyme involved in amino acid biosynthesis, CYR1, encoding adenylate cyclase necessary for cAMP production, and HSP31, encoding an enzyme involved in oxidative and DNA replication stress. None of these have been previously identified as interacting with ECM14, and it is difficult to identify a theme that might connect these to ECM14 function.

## Discussion

Fungi necessarily rely on a wide variety of hydrolases to break down and absorb the materials on which they grow. Few studies have examined carboxypeptidases expressed by fungi. To our knowledge, only two ascomycete metallocarboxypeptidases have been previously identified and characterized: several publications have examined one carboxypeptidase A (CPA) from *Metarhizium anisopliae* [37–39], while a second related CPA from *Trichophyton rubrum* had also been partially characterized [40]. Both enzymes exhibited typical CPA-like substrate specificity.

It is likely that Ecm14 is an inactive enzyme, or pseudoenzyme. Although it is difficult, if not impossible, to prove the absence of a function, we have been unable to detect carboxypeptidase activity in *S. cerevisiae* Ecm14 using standard chromogenic assays, and this is consistent with the substitution of critical residues within the pseudoactive site of Ecm14 (Fig. 1).

*ECM14* was previously identified through transposon mutagenesis as a gene providing resistance to calcofluor white and thus implicated in cell wall maintenance [33]. The *ecm14* mutant, in addition to being sensitive to calcofluor white, exhibited an increased mannose:glucose ratio and was sensitive to the effects of the aminoglycoside antibiotic hygromycin B and the glycolipid papulacandin B, thought to inhibit *β*1,3-glucan synthesis [33]. In this study of cell wall maintenance, two yeast strains with differing sensitivity to calcofluor white were used, and the *ecm14* mutant was only identified in one of these two screens. This differing sensitivity is consistent with our observation of strain-specific sensitivities, as mild calcofluor white sensitivity was only detected in BY4741a *ecm14*Δ cells grown long-term in one of two labs.

An understanding of the subcellular or extracellular location of a protein such as Ecm14 can provide useful information regarding the biological function of the protein. In our study, although we were unable to identify Ecm14 in the liquid growth media of *S. cerevisiae* cells expressing Ecm14-His6 (not shown), Ecm14-His6 was secreted when expressed heterologously from Sf9 cells (see Additional file 3: Figure S1). Others have shown differing results regarding localization. One large-scale analysis of yeast GFP-fusion proteins found that Ecm14-GFP was localized to the yeast vacuole [41]. When the SPELL (Serial Pattern of Expression Levels Locator) search engine [42] is queried for genes with expression profiles similar to *ECM14*, the top hits indicate GO term enrichment for the vacuole. A proteomic analysis of *S. cerevisiae* grown in biofilm-like mats identified Ecm14 as one of many proteins found in the extracellular matrix, but not secreted into the medium of a liquid culture [43]. These results suggest that Ecm14 likely functions within the extracellular matrix of yeast, maybe under biofilm-like conditions primarily, or possibly within the vacuole for the intracellular regulation of cell wall and extracellular matrix components.

Ecm14 has been identified in studies of several pathogenic fungi in recent years, and these studies continue to point toward a function in cell wall/extracellular matrix remodeling. A *C. albicans* screen for genes involved in aggregate invasive growth, a process related to biofilm formation, identified an *ECM14* mutant showing fewer aggregates than the wild-type [44]. This hints at a role in aggregate invasion, although the equivalent experiment performed in *S. cerevisiae* of the Σ1278b background, which can undergo aggregate invasive growth, did not result in a phenotype [44], suggesting either a false positive in the first screen or the use of inappropriate conditions for this assay with *S. cerevisiae*. A transcriptomics study of the plant pathogen *Verticillium dahliae* showed that the expression of Ecm14, along with a variety of cell wall degrading hydrolases, was upregulated in response to exudates from *V. dahliae*-susceptible cotton roots [45]. This again points to a possible role for Ecm14 in invasion. Finally, a recent analysis of glycosylation in the plant pathogen *M. oryzae* showed that the glycosylation of Ecm14 is upregulated primarily during mycelial growth as compared with the conidia and appressoria stages [46]. It was proposed that many proteins differentially glycosylated during mycelial growth were involved in cell wall biogenesis and nutrient utilization [46]. Our study found that Ecm14 expressed in *S. cerevisiae* was also glycosylated, although we did not examine the function of this modification (Fig. 3).

The molecular details of fungal invasion are important to understand as we try to counteract the pathogenic effects of many fungi. The above studies indicating a role for Ecm14 in fungal growth and pathogenesis lend support to our study in which we show that Ecm14 is highly conserved across the ascomycete lineage (see Fig. 2). An analysis of all reported interactions with *ECM14* supports the likelihood that a functional secretory pathway and other aspects of organellar trafficking are required for Ecm14 function. Unfortunately, our specific attempts to further identify redundant pathways through a synthetic lethal approach suggested that redundant pathways were many, such that we were unable to isolate a single-gene synthetic lethal clone from the analysis of nearly 30,000 mutants, or that Ecm14 was not required in the conditions and cells examined in this synthetic lethal assay. It could be that a clear phenotype for *S. cerevisiae ecm14*Δ mutants has not yet been identified because most yeast that are used in the lab are unable to effectively form aggregates. Scientists have effectively selected against this phenotype to make yeast genetics simpler.

## Conclusions

Our data suggest that, while Ecm14 may not be required in many laboratory strains of *S. cerevisiae*, Ecm14 is a conserved and important pseudoenzyme within many fungal species, that exists in zymogen form until activated by a trypsin-like endopeptidase, whereupon it likely functions in cell wall maintenance and/or fungal invasion. Notably, two vertebrate pseudoenzymes within the M14 MCP family are also involved in extracellular matrix functions, recently shown to bind collagen and support the mechanical strength of connective tissues [31, 47].

## Methods

Bioinformatics – BLASTp was used to search for all fungal proteins with homology to bovine CPA1. This dataset was further trimmed to remove short sequences and a Python script used to edit FASTA names. Clustal Omega [48] was used to create multiple alignments to enable analysis of active site residues and Jalview [49] was used for presentation of alignments. MEGA-X [50] was used to find the best-fit amino acid substitution model (WAG+G + F for our dataset), and this substitution model was used in IQ-TREE [51] to infer the best maximum likelihood phylogenetic tree. Branch supports were determined with an ultrafast bootstrap approximation with 1000 replicates in IQ-TREE. The SH-like approximate likelihood ratio test (aLRT) was also run with 1000 bootstrap replicates. Dendroscope [52] was used for presentation of the resulting consensus phylogenetic tree with bootstrap support values.

Structural modeling – The structural model for Ecm14 was constructed using SWISS-MODEL [53], with bovine procarboxypeptidase A1 (1PYT chain b) as template. Images were prepared with Pymol.

Baculovirus packaging – Plasmid DNA (pVL1392-Ecm14-His6; 50 ng) was mixed with 2.5 μl ProGreen baculovirus DNA (AB Vector) and water to a total volume of 25 μl. Profectin (AB Vector; 2.5 μl) was mixed with 22.5 μl water and added to the DNA mixture. Following a 20 min incubation, this was added to Sf9 cells (ATCC CRL-1711), seeded the day before in a 25-well dish at 2.5 × 10^5^ cells per well in 250 μl Sf-900™ II serum-free media (Thermo Fisher). Cells were checked daily for GFP fluorescence to confirm infection. Virus titer was amplified through several rounds of Sf9 infection to get a P3 stock.

Protein purification – Sf9 cells were infected at 2 × 10^6^ cells/ml with P3 baculovirus stock at 1% of the total volume. Conditioned media (typically 200–300 ml) was collected 3 days post-infection. Trypsin was added at 0.5 μg/ml and incubated at room temperature (22 °C) for 15 min to cleave the Ecm14 prodomain. The trypsin reaction was stopped by the addition of 1 mM phenylmethylsulfonyl fluoride (PMSF). A 1 ml bed volume of TALON metal affinity resin (Clontech) was washed twice with 10 ml wash buffer (50 mM sodium phosphate, 300 mM NaCl, pH 6.9) and added to conditioned media to which an additional 200 mM NaCl had been added. This was incubated at room temperature on a rocker for 45 min, followed by a short centrifugation to collect resin. The resin was washed twice in 10 ml wash buffer, rocking for 10 min, then transferred to a column and further washed by gravity with 15 ml wash buffer. Elution was performed by gravity with 5 ml wash buffer containing 300 mM imidazole, collecting 0.5 ml fractions. Purified protein was quantified by Bradford assay (Bio-Rad), and purity assessed by SDS-PAGE followed by staining with Bio-Safe™ Coomassie Stain (Bio-Rad).

Western blotting – Proteins were resolved by SDS-PAGE on 10% gels and transferred to nitrocellulose. Western blotting was performed according to standard protocol with mouse anti-His6 primary antibody (ThermoFisher, Clone HIS.H8; 1:2000 dilution) and horseradish peroxidase-conjugated secondary antibody (Cell Signaling Technology, 1:2000 dilution). Images were obtained using LumiGLO chemiluminescent reagent (Cell Signaling Technology).

Glycosylation analysis – Protein extracts were incubated with EndoH (NEB) in denaturing buffer and G5 reaction buffer for 1 h at 37 °C.

Carboxypeptidase enzyme assay – Ten to twenty microliters of protein extract or purified protein were incubated in a polystyrene 96-well plate with 0.5 mM substrate (3-(2-Furyl)acryloyl-Phe-Phe, −Phe-Ala, or -Glu-Glu; Bachem) in 50 mM Tris, 50 mM NaAc, 150 mM NaCl, at pH 5.5 to 7.5. Absorbance at 340 nm was read every minute for 30–60 min at 37 °C.

Edman degradation – Purified Ecm14-His6 (200 pmol) was resolved by SDS-PAGE, transferred to polyvinylidene difluoride membrane, and stained with Ponceau S. Bands were cut out and membranes destained with water. Six rounds of Edman degradation were performed by the Synthesis & Sequencing Facility at Johns Hopkins University.

Yeast culture – YPD media (10 g/l yeast extract, 20 g/l peptone, 20 g/l dextrose) was used for the routine propagation of yeast, supplemented when necessary with 8 ml/l of 0.5% (w/v) adenine (YPD + Ade), filter-sterilized Geneticin® (G418 sulfate, 500 μg/ml), or filter-sterilized nourseothricin sulfate (NTC, 100 μg/ml). Synthetic complete (SC-Ura) media (1% succinic acid, 0.6% sodium hydroxide, 0.67% yeast nitrogenous base, 1% casamino acids, 0.005% adenine, 0.002% tryptophan, 2% dextrose) was used for plasmid selection. When necessary, 0.001% uracil and 5-Fluoroorotic Acid (5FOA, 100X from Zymo Research) were added to SC media media. YPGal media (10 g/l yeast extract, 20 g/l peptone, 20 g/l galactose) was used for the destabilization of yeast centromere plasmids under the influence of the galactose promoter.

Yeast strains – For most early experiments, BY4741 (ATCC # 201388; *MAT*a (*ura3–52 lys2–801 ade2*–*101 trpl-1 his3–200 TUB4::3XHA*, *URA3*), a generous gift from Melanie Dobson, Dalhousie University) yeast were used. For the synthetic lethal experiment, *ecm14*Δ knockouts (*ecm14::KanMX*) were made in the AF-1A or AF-1D background (a modified W303 strain, ATCC #208352 and 201,238; *MAT*α and *MAT*a, respectively; *ade2–1 ura3–1 leu2–3,-112 his3–11,-15 trp1–1 ade3*∆; a generous gift from Melanie Dobson, Dalhousie University) by homologous recombination with the G418 cassette amplified from *ecm14*Δ cells of the yeast knockout collection (standard yeast transformation procedures) by PCR. Results were confirmed by PCR with ECM14_A, ECM14_D, and Kan_C primers.

Synthetic lethal assay – Fresh monoclonal colonies of AF-1D *ecm14::KanMX* [pSLS1-ECM14] were used to inoculate 10 ml of SC-Ura media. Following overnight culture, 2 × 10^8^ cells were pelleted, washed twice in 1 ml of sterile water, and resuspended in 1.5 ml of sterile sodium phosphate buffer (0.1 M, pH 7.0). Ethyl methanesulfonate (EMS) mutagenesis was performed by combining 0.7 ml of cell suspension, 1 ml of sodium phosphate buffer, and 50 μl of EMS in a sterile 15-ml polypropylene culture tube and incubating at 30 °C for 45–60 min. The remaining cell suspension was saved on ice as a pre-mutagenesis control. Mutagenesis was halted by adding 0.2 ml of EMS treated cells to a sterile culture tube containing 8 ml of sterile sodium thiosulfate solution (5%). Nonmutagenized control cells were diluted equivalently as above by substituting EMS with sterile water. Both EMS-treated and untreated cell suspensions were serially diluted to 1–3 viable cells/μl for plating on various growth media (100–300 viable cells per plate). The EMS mutagenesis protocol used in this study was adapted from Winston [54] and Barbour and Xiao [55].

Yeast sporulation – 1.5 ml 1% YPA (1% yeast extract, 2% peptone, 1% KAc) was inoculated with a single diploid colony and grown in 15 ml culture tubes shaking ~ 20 h at 30 °C. Cells were pelleted, washed with sterile water, and 1.5 ml sporulation media (1% KAc) added. Yeast were incubated at 250 rpm, 30 °C, for 7–10 days to sporulate. Two μl of zymolyase (Zymo Research) was added to 100 μl of the sporulation culture for 10 min, which was then diluted and plated following vigorous vortexing. As diploids do not maintain the plasmid on YPGal, red colonies indicated haploid cells.

Plasmids – See Table S2 for a list of plasmids used in this study. Plasmids were purified either using a Qiagen miniprep kit according to the manufacturer’s instructions, or by the nonionic detergent method [56]. In order to make pSLS-ECM14, the *ECM14* sequence was amplified by PCR with Platinum SuperFi® DNA Polymerase (Invitrogen) from BY4741a genomic DNA using ECM14_A and ECM14_D primers with included BamHI restriction sites for cloning into pSLS1. The insert was obtained in both orientations; in this study, all experiments used the minus orientation in which the insert is transcribed in the opposite direction of the *GAL1* promoter (see Additional file 3: Figure S4). Sequence was verified by Sanger sequencing (Genscript).

Whole-genome sequencing (WGS) and analysis – Fresh colonies from synthetic lethal mutants were cultured overnight at 30 °C with shaking in 3 ml of YEPD+Ade. Yeast were pelleted, washed with sterile water, and shipped on ice to The Sequencing Center in Fort Collins, Colorado. Whole-genome sequencing was performed using the Illumina MiniSeq® platform with a targeted mean coverage of ≈40–50×. Because the fully annotated yeast genome is S288C, and our yeast were of a W303 background, genome annotations were transferred from each S288C chromosome to the corresponding W303 chromosomal sequences recently produced by PacBio® sequencing (LYZE01000001–21; 47.0× coverage) by Matheson et al. [57]. This annotation was done using the BLAST-like “Annotate From …” alignment tool in Geneious Prime 2019.2.3. Annotations were matched using a generous similarity index of 80%. Paired reads from WGS were mapped against this annotated W303 genome using the Geneious Prime “Map to Reference” tool, and variants called. Automatically called variants were manually analyzed to eliminate variants conserved in our wild-type control, variants in non-coding sequences, and variants within regions of poor sequencing coverage.

## Supplementary Information

**Additional file 1.** Consensus phylogenetic tree.

**Additional file 2. **Maximum likelihood phylogenetic tree.

**Additional file 3: **Supporting information. **Table S1.** Whole-genome sequencing using the Illumina MiniSeq® platform. **Table S2.** Plasmids constructed and used in this study. **Figure S1.** Expression and purification of Ecm14. **Figure S2.** Edman degradation spectra. **Figure S3.** Large-scale screens identifying phenotypes for ECM14 mutants are not reproducible. **Figure S4.** Map of pSLS1-ECM14. **Figure S5.** The effect of EMS exposure time on yeast cell viability. **Figure S6.** Putative synthetic lethal mutants have reduced fitness on minimal media.

**Additional file 4.** WGS Variant Analysis.

**Additional file 5.** Gene Ontology (GO) Data.

**Additional file 6. **Original unmodified western blots.

## Data Availability

Whole genome sequencing data has been deposited in the Sequence Read Archive (SRA) at NCBI, with accession numbers SAMN15579334, SAMN15579335, SAMN15579336, SAMN15579337, SAMN15579338, SAMN15579339, SAMN15579340, SAMN15579341. All other data generated or analysed during this study are included in this published article [and its supplementary information files].

## Abbreviations

MCP: Metallocarboxypeptidases
VRK3: Vaccinia-related kinase 3
ERK: Extracellular-regulated kinase
Cpxm1: Carboxypeptidase X1
Cpxm2: Carboxypeptidase X2
Aebp1: AE-binding protein 1
CPD: Carboxypeptidase D
Ecm14: Extracellular mutant 14
BLAST: Basic local alignment search tool
Phe: Phenylalanine
Ala: Alanine
Glu: Glutamate
EMS: Ethyl methanesulfonate
CFW: Calcofluor white
YPD: Yeast extract peptone dextrose
YPGal: Yeast extract, peptone, galactose
SC: Synthetic complete
SGD: Saccharomyces genome database
GO: Gene Ontology
CPA: Carboxypeptidase A
GFP: Green fluorescent protein
PMSF: Phenylmethylsulfonyl fluoride
SDS-PAGE: Sodium dodecyl sulfate–polyacrylamide gel electrophoresis
WGS: Whole-genome sequencing
