# Supplementary material for: Biochemical and genetic analysis of Ecm14, a conserved fungal pseudopeptidase
Source: BMC Mol Cell Biol. 2020 Nov 30;21:86. doi: 10.1186/s12860-020-00330-w (PMC7706225; doi:10.1186/s12860-020-00330-w)
Supplement: Supplementary file 3 — Additional file 3: Supporting information. Table S1. Whole-genome sequencing using the Illumina MiniSeq® platform. Table S2. Plasmids constructed and used in this study. Figure S1. Expression and purification of Ecm14. Figure S2. Edman degradation spectra. Figure S3. Large-scale screens identifying phenotypes for ECM14 mutants are not reproducible. Figure S4. Map of pSLS1-ECM14. Figure S5. The effect of EMS exposure time on yeast cell viability. Figure S6. Putative synthetic lethal mutants have reduced fitness on minimal media. [file 12860_2020_330_MOESM3_ESM.pdf]

Supporting Information

Biochemical and genetic analysis of Ecm14, a conserved fungal pseudopeptidase

R. Christian McDonald, Matthew J. Schott, Temitope Idowu, and Peter J. Lyons

Materials Included:

1. Table S1. Whole-genome sequencing using the Illumina MiniSeq® platform.
2. Table S2. Plasmids constructed and used in this study.
3. Figure S1. Expression and purification of Ecm14.
4. Figure S2. Edman degradation spectra.
5. Figure S3. Large-scale screens identifying phenotypes for ECM14 mutants are not reproducible.
6. Figure S4. Map of pSLS1-ECM14
7. Figure S5. The effect of EMS exposure time on yeast cell viability.
8. Figure S6. Putative synthetic lethal mutants have reduced fitness on minimal media.
9. Excel Spreadsheet: WGS Variant Analysis
10. Excel Spreadsheet: Gene Ontology (GO) Data

Table S1. Whole-genome sequencing using the Illumina MiniSeq® platform.

Sample	Bases Read	Paired Reads	Matched Reads	Read Length x̄ (σ)	Coverage x̄	GC %
**WT**	878,377,752	5,997,874	4,899,419 (81.7%)	146.4 (15.7)	56.818	41.0
**SL6**	581,444,805	3,959,960	3,216,531 (80.6%)	146.8 (15.7)	36.873	41.0
**SL7**	494,730,687	3,451,230	2,746,783 (79.6%)	143.3 (19.6)	31.561	40.8
**SL8**	634,384,283	4,321,856	3,626,739 (83.9%)	146.8 (15.3)	42.510	40.5
**SL11**	633,162,416	4,309,744	3,721,436 (86.3%)	146.9 (15.2)	43.498	40.4
**SL12**	743,482,836	5,087,184	4,611498 (90.6%)	146.1 (16.2)	53.302	40.4
**SL13**	618,472,666	4,215,912	3,744,904 (88.8%)	146.7 (16.1)	44.315	40.5
**SL14**	561,659,729	3,869,674	3,400,631 (87.9%)	145.1 (17.9)	39.176	40.6
**Σ**	**5,145,715,174**	**35,213,434**	**29,967,941 (85.1%)**			

Table S2. Plasmids constructed and used in this study.

Name	Kb	Insert	Features	Source	Reference
pRS316	4.9	--	Amp^R, CEN4/ARS1, LacZα, ORI, URA3	Melanie J. Dobson	Sikorski and Hieter, 1989
pAG25	3.7	--	Amp^R, NatMX, ORI	Addgene	Goldstein et al., 1999
pSLS1	10.8	--	ADE3, Amp^R, CEN4/ARS1, ORI, URA3, pGAL1	Melanie J. Dobson	Barbour et al., 2000
pRS316-NatMX	6.2	NatMX	Amp^R, CEN4/ARS1, LacZα, ORI, URA3	This study	--
pBluescript-ECM14*	4.8	ECM14	Amp^R, LacZα, ORI	This study	--
pSLS1-ECM14*	12.7	ECM14	ADE3, Amp^R, CEN4/ARS1, ORI, URA3, pGAL1	This study	--

* Both insert orientations were constructed. The minus orientation was used in this study.

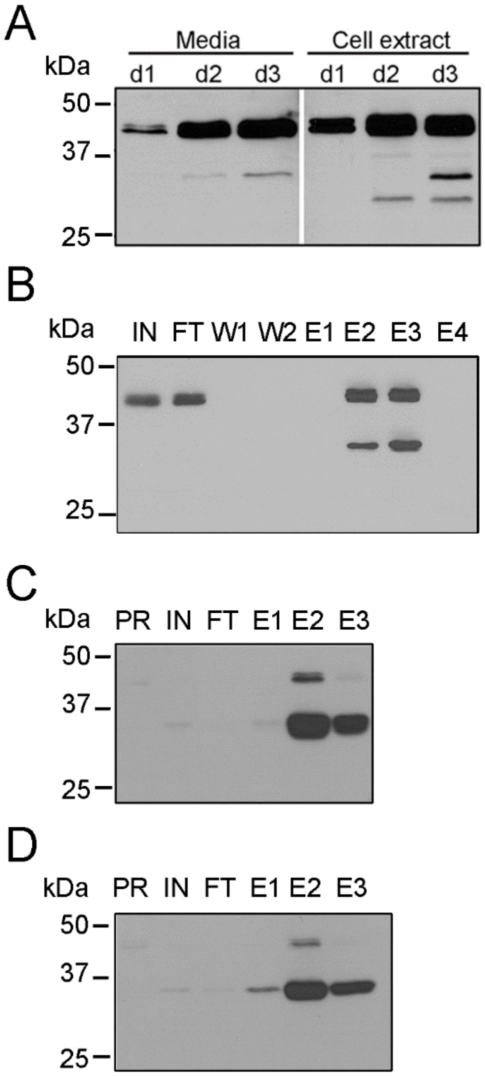

Figure S1. Expression and purification of Ecm14. A) About 50% of Ecm14-His6 could be collected from the serum-free Sf9 media. Sf9 cells were infected with high-titer virus baculovirus stock. A portion of the media and cells were collected following 1, 2, or 3 days of infection. A volume of media or cell extract equivalent to 0.1 % of the media or 0.125% of the cells was analyzed by Western blotting with an anti-His6 antibody. B) 100 ml of conditioned media was incubated with cobalt-metal affinity resin to purify Ecm14-His6. Equal volumes of input (IN), flow-through (FT), washes (W1 and W2), and 0.5 ml elution fractions (E1-E4) were analyzed by western blotting with an anti-His6 antibody. Very little Ecm14-His6 bound to the resin, as indicated by similar band intensity of input and flow-through. Note that although the majority of input Ecm14-His6 is in the proform (45 kDa), a large portion of the elute is in the mature form (35 kDa), suggesting that this preferentially binds. When 100 ml conditioned media was first incubated with 0.5 ug/ul chymotrypsin (60 min at 20°C; C) or with 0.5 ug/ul trypsin (10 min at 20°C; D), the majority of Ecm14-His6 bound (compare IN and FT) and was eluted from the column. PR indicates conditioned media before digestion with chymotrypsin or trypsin.

A

Standard

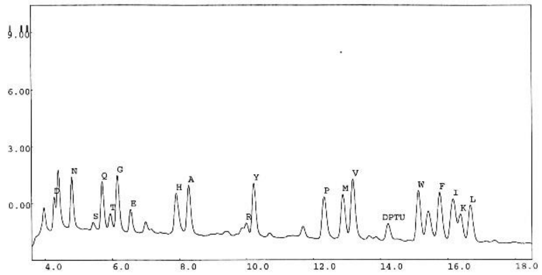

B

Ecm14 cut with trypsin (AA1)

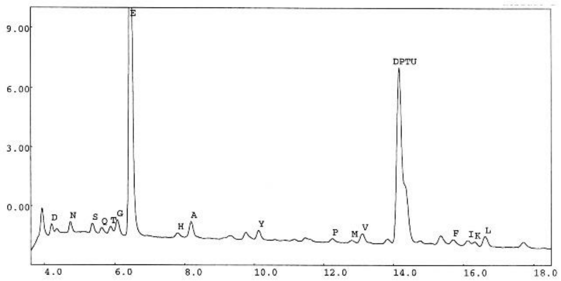

Ecm14 cut with trypsin (AA2)

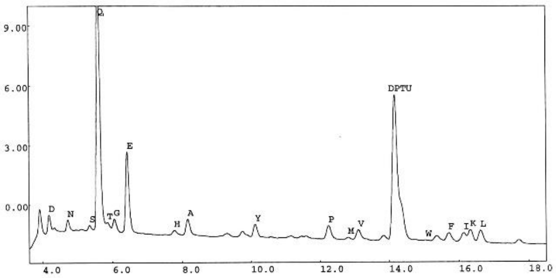

Ecm14 cut with trypsin (AA3)

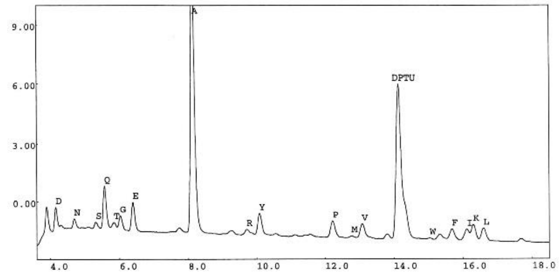

Ecm14 cut with trypsin (AA4)

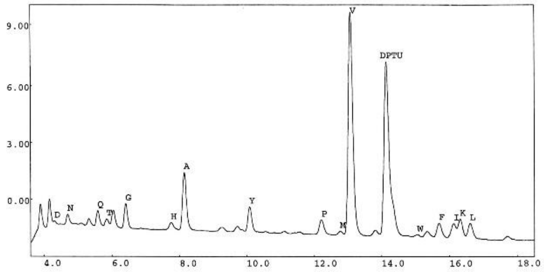

Ecm14 cut with trypsin (AA5)

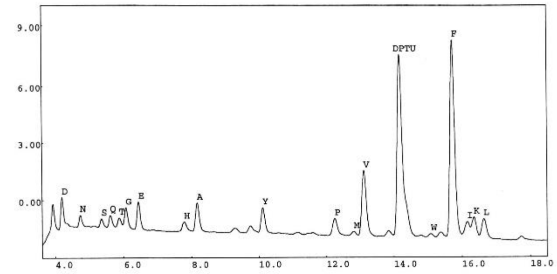

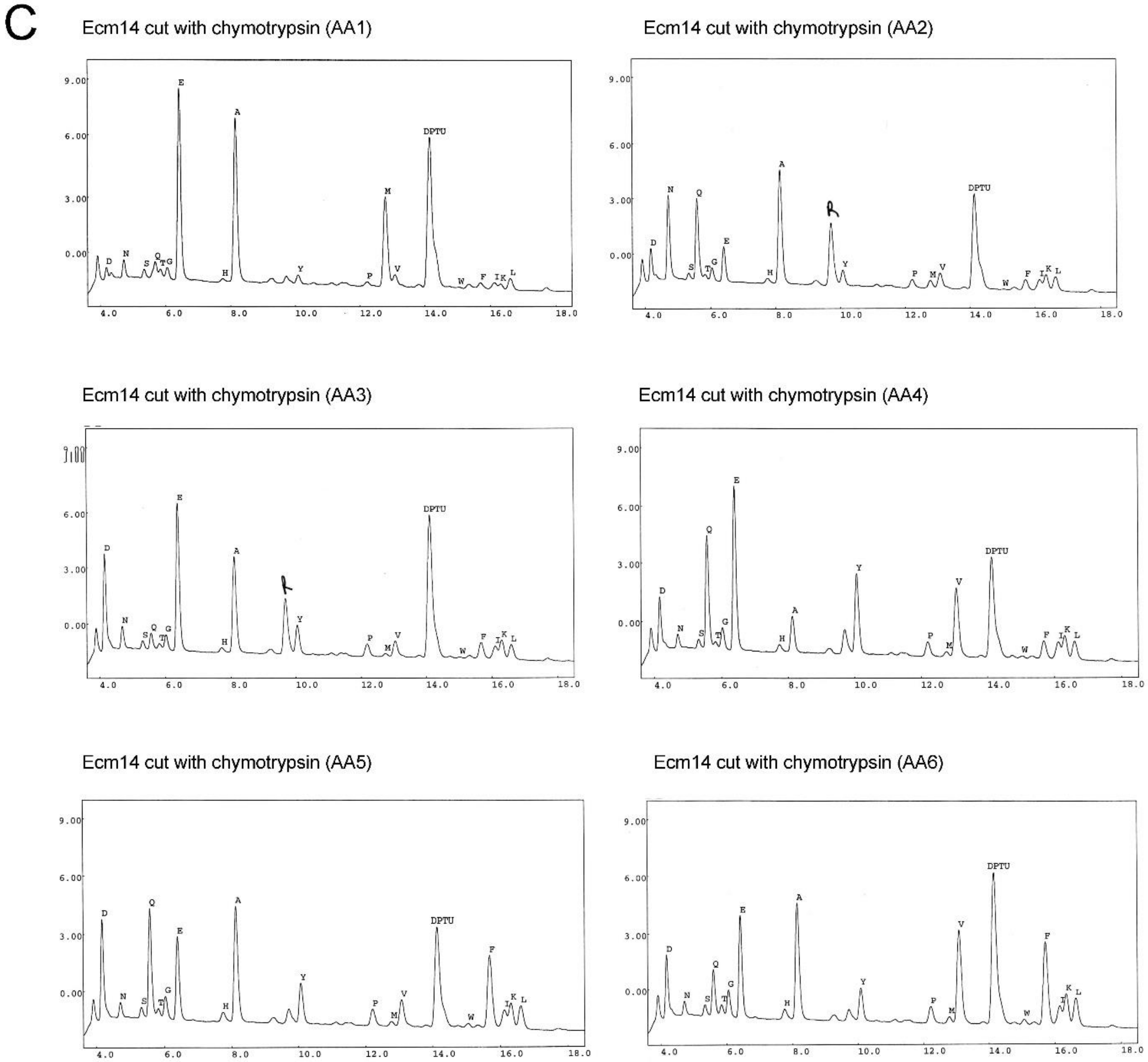

Figure S2. Edman degradation spectra.

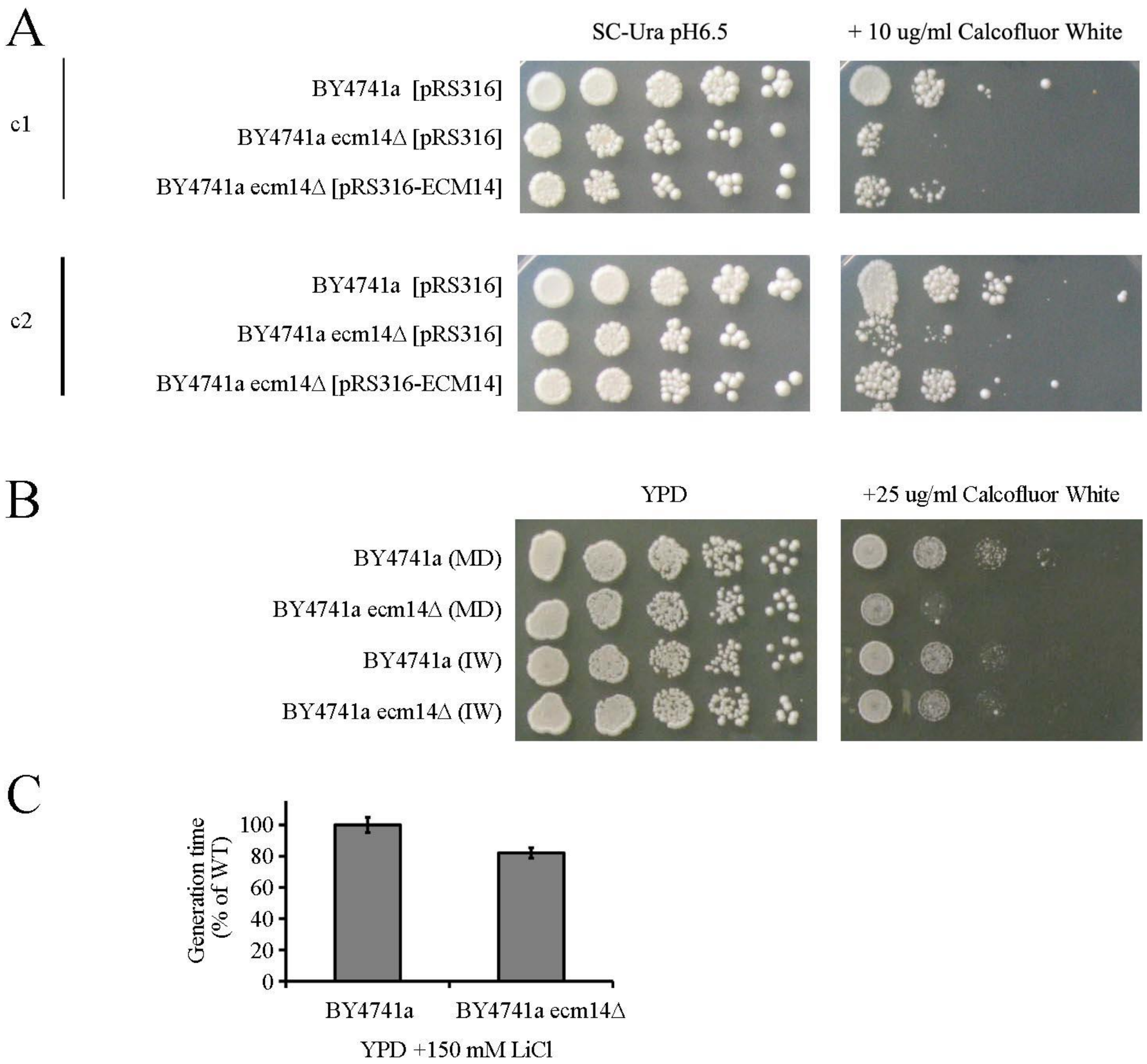

Figure S3. Large-scale screens identifying phenotypes for ECM14 mutants are not reproducible. A) Five-fold dilutions of the indicated yeast transformed with the pRS316 plasmid as control, or the pRS16 plasmid expressing ECM14, were spotted onto SC-Ura plates +/- calcofluor white. Two clones (c1, c2) of each transformant were spotted, showing similar results. B) Five-fold dilutions of BY4741a yeast from two different labs (MD and IW) were spotted onto YPD plates +/- calcofluor white. While the MD yeast indicated mild calcofluor white sensitivity, the EW yeast did not. C) Yeast were grown in liquid YPD medium + 150 mM LiCl. Cell density (OD660) was measured at intervals. Error bars indicate standard deviation (n=3; p = 0.011).

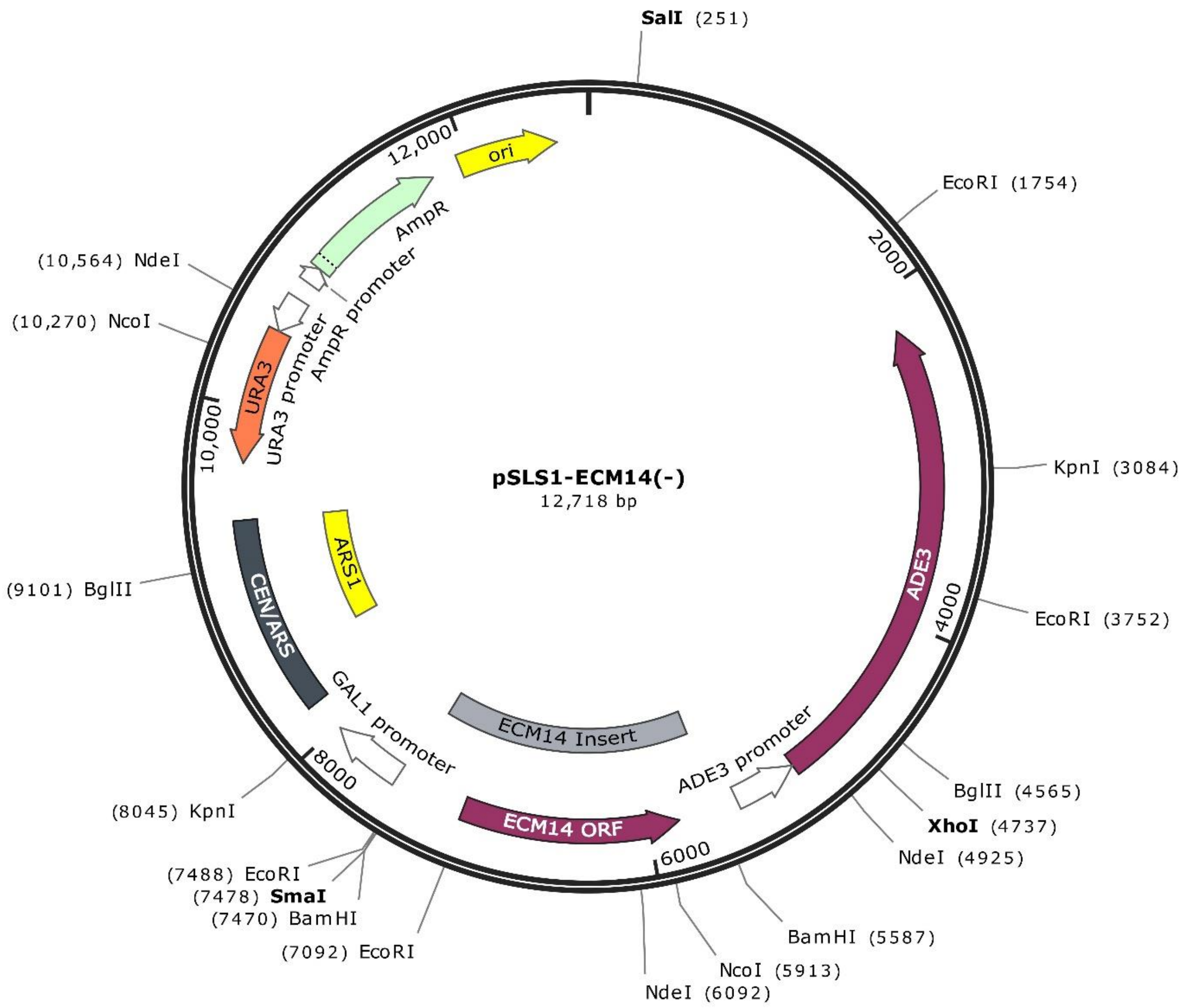

Figure S4. Map of pSLS1-ECM14. This (-) orientation was used in all experiments in this study.

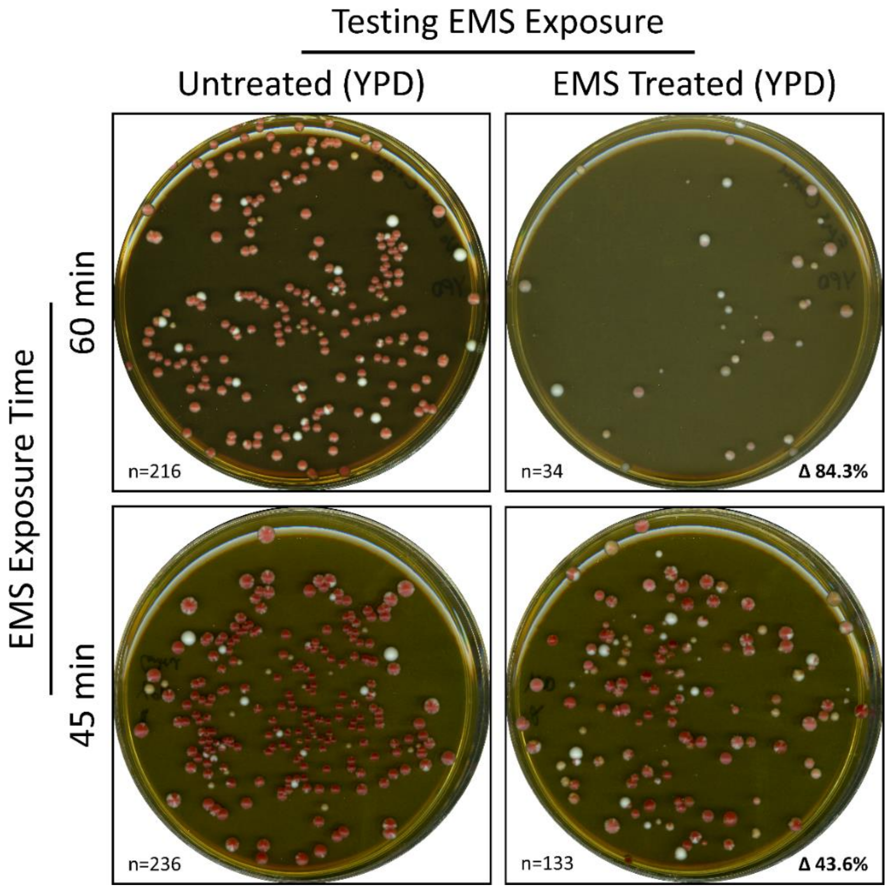

Figure S5. The effect of EMS exposure time on yeast cell viability. EMS-treated and untreated cells were plated on YPD media to test the effect of EMS exposure at both 60 minutes and 45 minutes. Sixty minutes of exposure was ineffective at producing enough viable yeast cells per plate (84.3% cell death). Reducing the EMS exposure to 45 minutes was determined to be more effective at generating a more ideal cell death (43.6%) in the yeast strains used in this study.

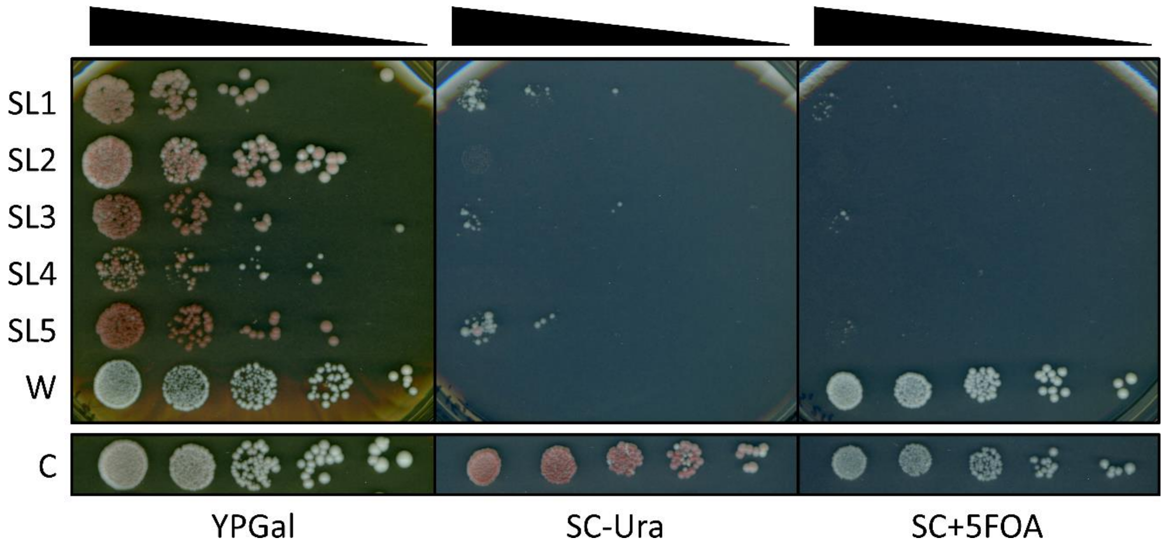

Figure S6. Putative synthetic lethal mutants have reduced fitness on minimal media. Spotting assay on YPGal, SC-Ura, and SC+5FOA. Serial 1:10 dilutions of five putative synthetic lethal mutants (**SL1-5**), one EMS-treated white, non-synthetic lethal colony (**W**), and one untreated control strain (**C**). Plates were incubated at 30°C for ≈120 hours. Putative synthetic lethal mutants were derived from EMS-treated ecm14Δ [pSLS1-ECM14] colonies initially screened on YPGal.
