# Supplementary material for: Biochemical and genetic analysis of Ecm14, a conserved fungal pseudopeptidase
Source: BMC Mol Cell Biol. 2020 Nov 30;21:86. doi: 10.1186/s12860-020-00330-w (PMC7706225; doi:10.1186/s12860-020-00330-w)
Supplement: Supplementary file 6 — Additional file 6. Original unmodified western blots. [file 12860_2020_330_MOESM6_ESM.pdf]

COMPLETE UNMODIFIED GELS/BLOTS

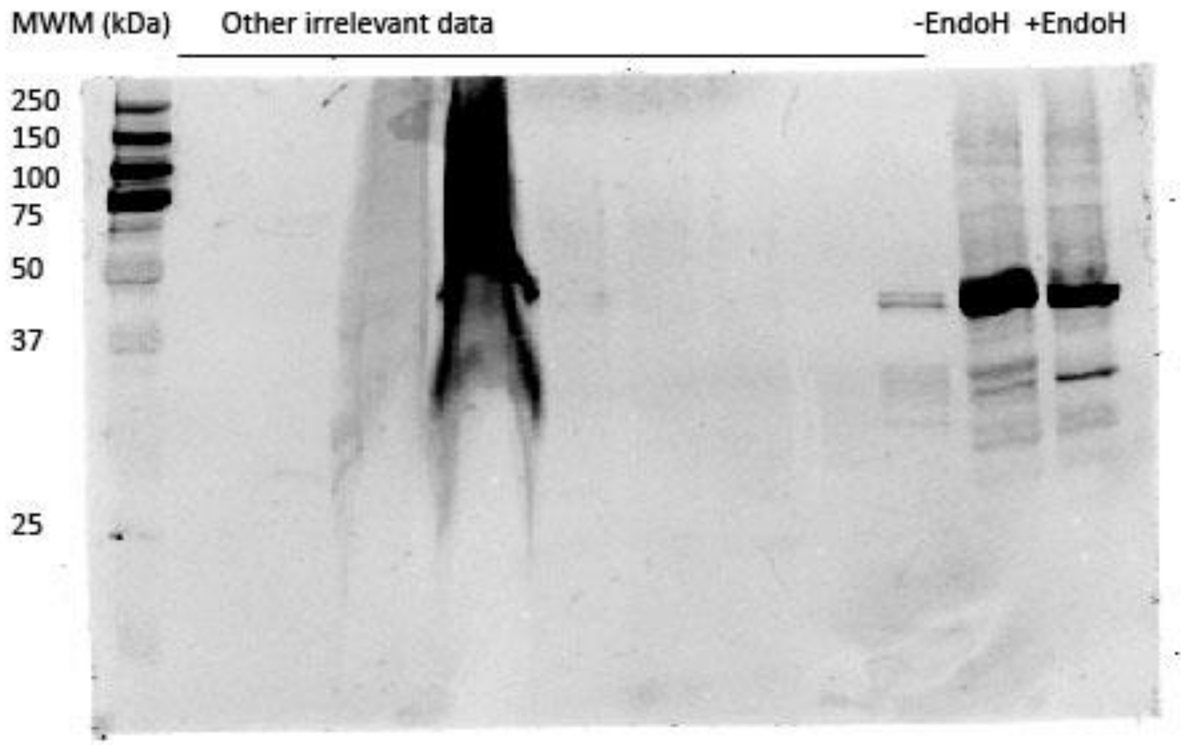

Figure 3a. Ecm14 is N-glycosylated and processed by an endopeptidase in S. cerevisiae. Yeast extracts that overexpressed Ecm14-His6 were incubated with endoglycosidase H (EndoH) and analyzed by western blotting with an anti-His6 antibody. ProEcm14 observed as a broad band at 45 kDa (likely two bands) collapses to one thin band with EndoH, and mature Ecm14 observed as two bands at 35 kDa collapses to one.

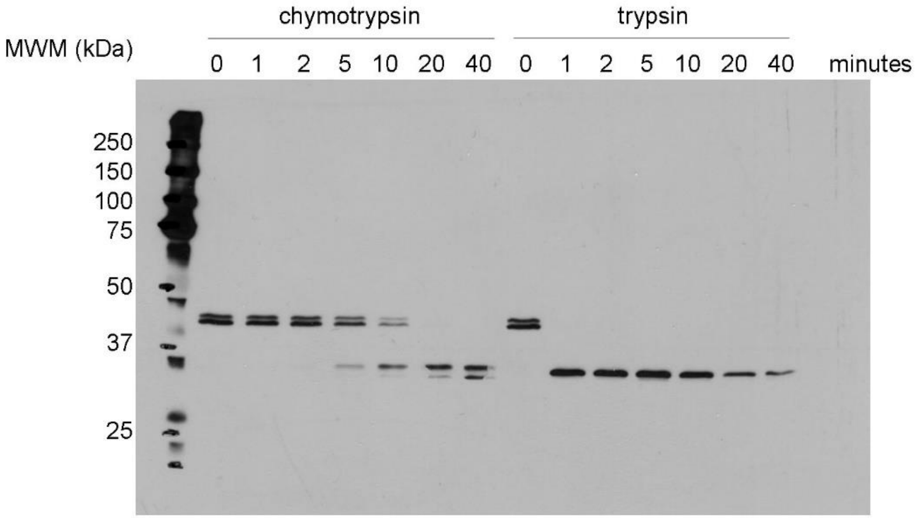

Figure 4a. The prodomain of Ecm14 can be cleaved by chymotrypsin and trypsin enzymes. Media containing proEcm14-His6 secreted from baculovirus infected Sf9 cells was digested with chymotrypsin or trypsin for the indicated times and analyzed by Western blotting with an anti-His6 antibody.

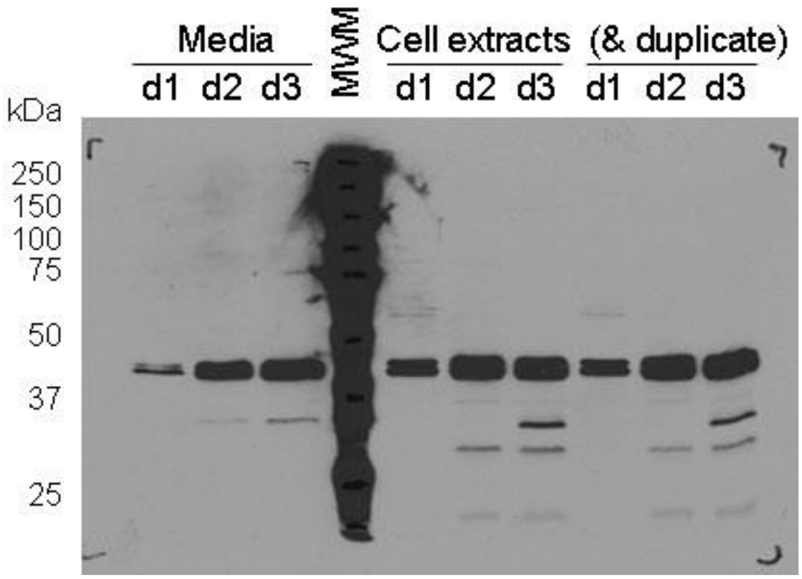

Figure S1a. Expression and purification of Ecm14. About 50% of Ecm14-His6 could be collected from the serum-free Sf9 media. Sf9 cells were infected with high-titer virus baculovirus stock. A portion of the media and cells were collected following 1, 2, or 3 days of infection. A volume of media or cell extract equivalent to 0.1 % of the media or 0.125% of the cells was analyzed by Western blotting with an anti-His6 antibody.

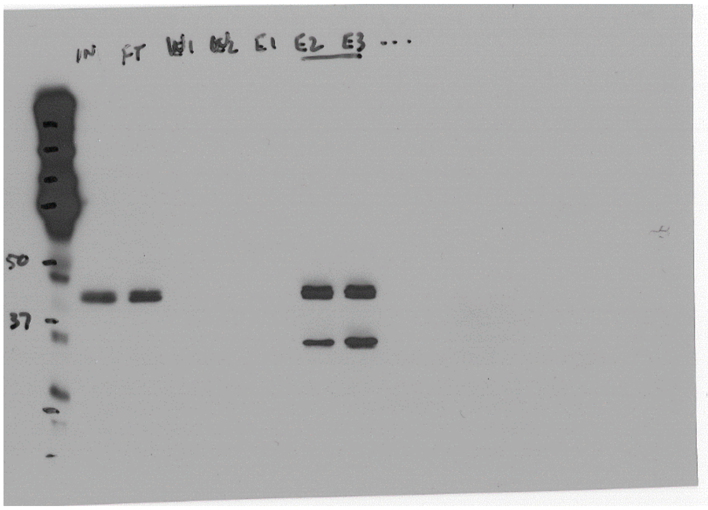

Figure S1b. Expression and purification of Ecm14. 100 ml of conditioned media was incubated with cobalt-metal affinity resin to purify Ecm14-His6. Equal volumes of input (IN), flow-through (FT), washes (W1 and W2), and 0.5 ml elution fractions (E1-E4) were analyzed by western blotting with an anti-His6 antibody. Very little Ecm14-His6 bound to the resin, as indicated by similar band intensity of input and flow-through. Note that although the majority of input Ecm14-His6 is in the proform (45 kDa), a large portion of the elute is in the mature form (35 kDa), suggesting that this preferentially binds.

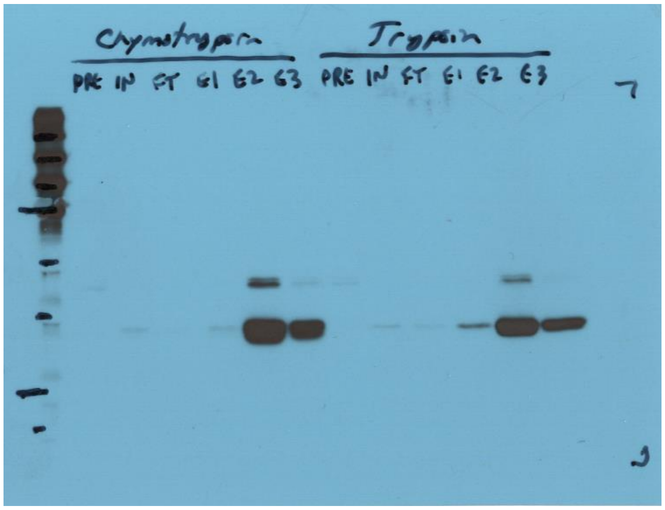

Figure S1c-d. Expression and purification of Ecm14. When 100 ml conditioned media was first incubated with 0.5 ug/ul chymotrypsin (60 min at 20°C; C) or with 0.5 ug/ul trypsin (10 min at 20°C; D), the majority of Ecm14-His6 bound (compare IN and FT) and was eluted from the column. PR indicates conditioned media before digestion with chymotrypsin or trypsin.
